# PLA Electrospun Fibers Reinforced with Organic and Inorganic Nanoparticles: A Comparative Study

**DOI:** 10.3390/molecules26164925

**Published:** 2021-08-14

**Authors:** Adrián Leonés, Valentina Salaris, Alicia Mujica-Garcia, Marina P. Arrieta, Daniel Lopez, Marcela Lieblich, José Maria Kenny, Laura Peponi

**Affiliations:** 1 Instituto de Ciencia y Tecnología de Polímeros (ICTP-CSIC), C/Juan de la Cierva 3, 28006 Madrid, Spain; aleones@ictp.csic.es (A.L.); v.salaris@ictp.csic.es (V.S.); aliciamujicagarcia@gmail.com (A.M.-G.); m.arrieta@upm.es (M.P.A.); daniel.l.g@csic.es (D.L.); 2 Interdisciplinary Platform for Sustainable Plastics towards a Circular Economy, The Spanish National Research Council (SusPlast-CSIC), 28006 Madrid, Spain; 3 Civil and Environmental Engineering Department and UDR INSTM, University of Perugia, Strada di Pentima 4, 05100 Terni, Italy; 4 Departamento de Ingeniería Química Industrial y del Medio Ambiente, Escuela Politécnica Superior de Ingenieros Industriales, Universidad Politécnica de Madrid (ETSII-UPM), Calle José Gutiérrez Abascal 2, 28006 Madrid, Spain; 5 Grupo de Investigación: Polímeros, Caracterización y Aplicaciones (POLCA), 28006 Madrid, Spain; 6 Centro Nacional de Investigaciones Metalúrgicas (CENIM-CSIC), 28040 Madrid, Spain; marcela@cenim.csic.es

**Keywords:** electrospinning, PLA, organic nanoparticles, inorganic nanoparticles

## Abstract

In this work, different poly (lactic acid) (PLA)-based nanocomposite electrospun fibers, reinforced with both organic and inorganic nanoparticles, were obtained. As organic fibers, cellulose nanocrystals, CNC, both neat and functionalized by “*grafting from*” reaction, chitosan and graphene were used; meanwhile, hydroxyapatite and silver nanoparticles were used as inorganic fibers. All of the nanoparticles were added at 1 wt% with respect to the PLA matrix in order to be able to compare their effect. The main aim of this work was to study the morphological, thermal and mechanical properties of the different systems, looking for differences between the effects of the addition of organic or inorganic nanoparticles. No differences were found in either the glass transition temperature or the melting temperature between the different electrospun systems. However, systems reinforced with both neat and functionalized CNC exhibited an enhanced degree of crystallinity of the electrospun fibers, by up to 12.3%. From a mechanical point of view, both organic and inorganic nanoparticles exhibited a decreased elastic modulus and tensile strength in comparison to neat electrospun PLA fibers, improving their elongation at break. Furthermore, all of the organic and inorganic reinforced systems disintegrated under composting conditions after 35 days.

## 1. Introduction

Poly(lactic acid) (PLA) is an aliphatic polyester that has gained great attention as an alternative material to conventional synthetic polymers. It is obtained from renewable agricultural raw materials, mainly starch and sugar, which are fermented to lactic acid [1,2]. Due to the presence of two chiral centers, two optical isomers l,l-lactide (l-LA) and d,d-lactide, can be obtained that are able to strongly influence the final properties of the polymer [1].

PLA is a biocompatible polymer that is widely used in several fields such as food packaging, biomedicine and textiles, among others [3,4,5]. Additionally, PLA shows hydrolytic degradation and is slowly broken into non-toxic metabolites [6]. However, when using PLA, its thermal and mechanical properties have to be improved in order to achieve good performance [7]. In particular, PLA shows good stiffness and strength and can be easily processed into different forms such as films, fibers and nanocomposite bulk materials [8,9,10]. However, its brittleness has to be improved by blending or copolymerizing it with more elastic polymers such as poly(ε-caprolactone), PCL [11,12,13].

To obtain PLA-based fibers with enhanced thermal and mechanical properties, melt-spinning or electrospinning can be used [9,14]. In particular, solution electrospinning is a simple, effective, versatile and low-cost technique that can be used to produce polymeric fibers through the application of a high electric voltage to a viscous polymer solution in a volatile solvent under ambient conditions [15,16]. The versatility of this technique makes it possible to easily obtain woven non-woven electrospun fibers, e-fibers, that can be used in many fields such as biomedicine, food packaging and agriculture, among others [17,18,19]. Moreover, once a good suspension of nanoparticles, NPs, has been obtained, they can be dispersed into the polymeric solution and then processed by electrospinning, resulting in nanoreinforced electrospun fiber mats, which possess improved properties with respect to neat PLA e-fibers [8,13,19]. The main aim of our work is the reinforcement of the PLA matrix using both organic and inorganic NPs. In particular, among organic NPs, cellulose nanocrystals, CNC, which are derived from microcrystalline cellulose, can be used, which is advantageous because cellulose, which can be obtained from renewable and sustainable resources [20,21], has been widely studied, and is the most abundant polysaccharide in the world [22]. From a chemical point of view, its anhydro-glucose rings are characterized by the presence of many functional pendant groups, facilitating chemical modifications such as “*grafting from*” modifications [23]. At the nano level, CNC can be obtained by the sulfuric acid hydrolysis of microcellulose, yielding nanoparticles with different morphologies at a high aspect ratio [24]. However, their hydrophilic nature may induce agglomeration when added to hydrophobic polymeric matrices. Therefore, this limitation needs to be overcome by modifying their surfaces by means of “*grafting from*” reactions [24,25,26].

The second-most abundant organic substance in the world is chitosan [27,28]. Chitosan has been widely studied in different fields, such as alcoholic fermentation, due to its role in metal chelation, and the clarification and reduction of contaminants in enology [29], in the food industry [30], or for microbial control [31]. Moreover, chitosan is one of the most important derivatives of chitin, and is obtained through the partial deacetylation of chitin [32]. Both chitin and chitosan are polymers that are biodegradable, biocompatible and non-toxic [33].

Furthermore, among organic NPs, graphene is considered to be one of the most promising for the reinforcement of polymeric matrices [34,35]. In fact, its chemical structure is based on carbon–carbon bonds, which provide stiffness, high thermal conductivity and remarkable mechanical properties [36]. Therefore, graphene can be used to produce polymeric nanocomposites characterized by enhanced properties, such as improved thermal and mechanical response [37,38]. Another example of organic carbon-based nanofillers used with PLA-based materials is carbon nanotubes, CNTs. CNTs are expected to provide a very high surface area for π-π stacking with polymer electrospun matrices, thus resulting in good adhesion [39].

On the other hand, the use of inorganic NPs to reinforce PLA-based materials can play a crucial role in biological process such as bone growth [40,41]. Hydroxyapatite, HA, a calcium phosphate salt, is present in living bone structures [42] and shows exceptional biocompatibility and bioactivity with respect to bone and tissue cells; as a result, it has been extensively studied for use as a reconstructive material in medical applications [43,44].

Silver nanoparticles, Ag NPs, are widely used in fields such as antibacterial textiles [45], polymer films for food packaging [46], and filters for water [47], among others. Moreover, Ag NPs are capable of releasing silver ions in a controlled manner, leading to a powerful antibacterial activity against a large number of bacteria [48,49].

Therefore, in this work, an extensive study of the use of different organic and inorganic NPs as nanofillers for PLA-based electrospun fibers is reported, with a particular focus on their different responses. Previously, we reported a study on the effect of the addition of both organic and inorganic NPs on PCL electrospun fiber mats; no considerable differences were found with the addition of organic and inorganic NPs, and there were no significant variations with respect to glass transition temperature, T_g_, melting temperature, T_m_, or degree of crystallinity, leading in all cases to electrospun mats with high crystallinity. From a mechanical point of view, the use of both organic and inorganic NPs with PCL resulted in electrospun mats with increased flexibility, possessing increased elongation at break [24]. Consequently, the main strategy of this work is to compare the effects of the addition of organic and inorganic NPs on the final properties of PLA-based electrospun materials. In particular, CNC, both neat and functionalized by chitosan, graphene, and by grafting PLLA chains onto the external surface (CNC-g-PLLA), are used as organic NPs, while HA and Ag NPs are used as inorganic ones. All of the NPs used are commercially available, with the exception of of CNC and CNC-g-PLLA, which were synthesized and modified in our lab. Moreover, all of the NPs were added at 1 wt% with respect to the PLA matrix in order to compare their effects, mainly with respect to their morphology, and their thermal and mechanical response. Firstly, the processing conditions were optimized with the aim of obtaining homogenous and defect-free PLA e-fibers. Then, PLA-based e-fibers were obtained with both organic and inorganic NPs using the same processing conditions. The morphological characterization was carried out by means of scanning electron microscopy, SEM. Moreover, thermal behavior and mechanical properties were studied and compared with those of the neat PLA e-fibers. Finally, electrospun mats were disintegrated under composting conditions with the objective of studying how the different NPs influence the compostability of PLA-based e-fibers.

## 2. Results and Discussion

The synthesis and functionalization of CNC was carried out in our lab in order to obtain PLA-based electrospun mats. Specifically, acid hydrolysis of commercial microcrystalline cellulose was performed to obtain cellulose nanocrystals, while in a second step, they were functionalized by means of the “*grafting from*” reaction. Specifically, the ring-opening polymerization of the PLA homopolymer prepared from optically pure l-LA, PLLA, was conducted in order to graft PLLA chains onto the CNC surface using the hydroxyl groups of the CNC as anchoring points. The synthesis and functionalization of the CNC are characterized in the Supplementary Materials. Briefly, vibrational analysis and thermogravimetric analysis, TGA, were performed for the purpose of studying the presence and the amount of PLLA on the CNC, as reported in Figure S1.

However, prior to the optimization of the electrospinning process, a morphological analysis of both organic and inorganic NPs was carried out. In agreement with our previous work [24], the FE-SEM images confirmed the size of the NPs to be in the nanometric range. Images of the different NPs, as well as their average dimensions, are presented in Figure S2. Specifically, CNC and CNC-g-PLLA possessed a rod-like morphology, with average lengths of 179 ± 20 nm and 298 ± 56 nm, respectively. Grafting PLLA chains onto the CNC surface increases both the average length and the average diameter of CNC-g-PLLA NPs by up to 40% with respect to neat CNC, increasing these values from 14.1 ± 1.6 nm for neat CNC to 17.7 ± 2.9 nm for CNC-g-PLLA.

Regarding the morphology of the commercial NPs, Ag, chitosan and HA, all of them exhibited a spherical morphology with average diameter values below 30 nm, specifically, 22.4 ± 2.4 nm, 23.7 ± 2.3 nm and 18.8 ± 2.0 nm, respectively, while graphene nanoplatelets exhibited a length of about 290 nm.

Therefore, after characterizing the morphology of the different NPs, the electrospinning process parameters were optimized in order to obtain homogeneous woven non-woven PLA-based e-fibers without defects. When working with electrospinning, it is necessary to consider two main classes of parameters: those related to the polymer solution and those related to the processing conditions further to the ambient conditions [19]. In order to optimize both parameters, they need to be varied and correlated with the morphology of the e-fibers, and in particular with their diameter. However, considering the numerous variables involved, a “one factor at time” method was adopted in this work, with the aim of finding the optimum window of the working conditions. Firstly, the morphologies of the fibers were correlated with the polymer concentration in the starting solutions. Then, once the optimum concentration was chosen, the best conditions for the flow rate were determined, and so on for the other parameters. The most relevant experimental variables used with respect to concentration, solvent and polymer flow rate, as well as the positive and negative applied voltage, are summarized in Table 1; obviously, many more experiments than those reported in Table 1 were performed in order to find the optimum working window.

The results show that to ensure the formation of fibers, a minimum concentration of polymer is required. In our system, PLA electrospinning commenced at a minimum concentration of 4 wt%, indicating that no e-fibers were obtained below this concentration [14]. In addition, as expected, by increasing the PLA concentration in the solution, fibers with larger diameters were obtained. Moreover, for the correct formation of the Taylor cone, the electric charges have to favor the formation of fibers from the polymer solution drops [50]. Regarding our results, by increasing the applied voltage, e-fibers with more homogenous diameter values were obtained. In summary, the best conditions obtained for our system were a concentration of PLA between 8 and 10 wt%, a low flow rate for both the polymer solution and the solvent, and application of a high voltage; specifically, the conditions studied in run PLA_46_. Therefore, once the optimal conditions for neat PLA e-fibers had been set, the PLA-based e-fibers were obtained following the same experimental procedure in order for comparisons among them to be possible. According to the scientific literature and our previous work [24], we set an amount of 1 wt% NPs with respect to the polymer, as this is a good concentration to disperse into the polymeric solution. Therefore, PLA-based e-fiber mats reinforced with CNC, CNC-g-PLLA, HA, Ag, chitosan and graphene NPs were successfully obtained and characterized.

**Figure 1:**
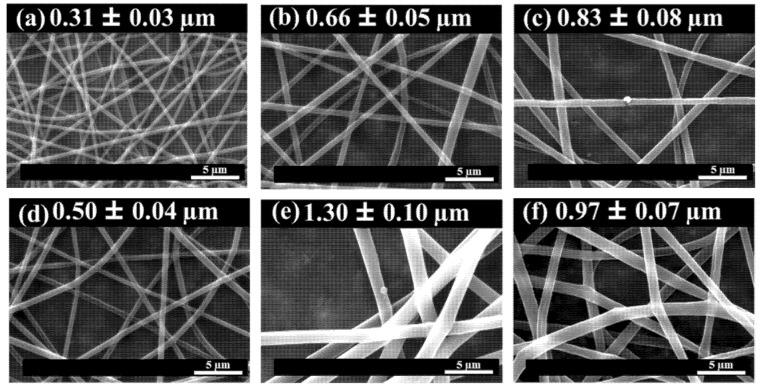
SEM images of (**a**) PLA/CNC, (**b**) PLA/CNC-g-PLLA, (**c**) PLA/Ag, (**d**) PLA/chitosan, (**e**) PLA/HA, (**f**) PLA/graphene e-fibers, as well as their corresponding average diameters.

The average diameter and the morphology of the PLA-based e-fibers were determined on the basis of SEM images. In Figure 1, the SEM images of the reinforced e-fibers are presented, while the SEM images of the neat PLA electrospun fibers are presented in the Supplementary Material as Figure S3. Moreover, their average diameters, calculated by ImageJ software on the basis of 50 measurements in different e-fibers as well as at different points of the same electrospun fiber, are reported in the figures.

From a morphological point of view, randomly oriented e-fibers in the micron range were obtained with all of the NPs studied. Moreover, no bead formation was reported in any of the different nanocomposite electrospun fibers, confirming that the working window set was suitable for obtaining smooth and homogenous e-fibers. For a deeper understanding of the effect of the variation of parameters on the morphology of the e-fibers, a one-way analysis of variance (ANOVA) for the diameters was carried out, as reported in Table 2.

From a statistical point of view, no significant differences in the average diameter values were obtained between the neat PLA e-fibers and the e-fibers reinforced with PLA/Ag (inorganic NPs) or PLA/graphene (organic NPs) (*p* < 0.05). The other PLA-based e-fibers presented statistically significant differences between them with respect to average diameter (*p* < 0.05). Specifically, when adding organic NPs such as CNC, CNC-g-PLLA or chitosan, there is a decreasing trend in terms of average diameter with respect to the neat PLA e-fibers. In contrast, with HA NPs, the average diameter increased to 1.30 ± 0.10 µm, representing about a 40% increase in the average diameter values with respect to the neat PLA e-fibers. The increase in the average diameter of electrospun fibers as a result of adding HA NPs was in accordance with previously reported results. Sonseca et al. reported an increase in the average diameter of electrospun PLA/HA fibers of 36% with respect to neat PLA e-fibers [44]. In addition, this behavior has also been observed in other polymer matrices, such as PCL. Leonés et al. reported an increase in the average diameter of PCL/HA fibers of 33% with respect to neat PCL e-fibers [24]. Moreover, there is a balance between the increase in viscosity and the increase in repulsive force resulting from the presence of NPs during electrospinning, which affects the diameter of the fibers [51].

In order to study the thermal responses of the different electrospun systems, DSC analysis was conducted; the thermal parameters obtained from the first heating scan are summarized in Table 3.

The characteristic T_g_ value of neat PLA e-fibers was observed at 55 °C, and similar values were observed in all of the PLA-based e-fibers. It is important to note that the addition of different NPs, both organic and inorganic, does not strongly change the glass transition temperature. The addition of chitosan slightly increased the T_g_ value to 60 °C, which is in accordance with results previously reported in the literature [52]. Chitosan is known to have very rigid structure, with a glass transition temperature of 203 °C, which can provoke an increase of T_g_ in PLA matrix [52].

PLA/CNC and PLA/CNC-g-PLLA e-fibers presented the highest degree of crystallinity, 12.3% in both samples, evidencing a good dispersion between the amorphous region of the PLA matrix and the crystalline NPs and their nucleating effect. In contrast, the lowest degree of crystallinity was observed in the PLA/chitosan e-fibers, where a degree of crystallinity of 5.4% was observed. The presence of chitosan may affect the formation of the crystalline structure of PLA during crystallization, resulting in low degrees of crystallinity. This behavior is in accordance with previous results obtained using chitosan in PLA matrix [52]. Regarding the other PLA-based e-fibers, in all cases, the degree of crystallinity slightly decreased compared to neat PLA e-fibers, regardless of whether the NPs added were organic or inorganic in nature.

Additionally, the thermal stability of PLA-based e-fibers was studied by means of thermogravimetric analysis. The maximum degradation temperature for each electrospun sample is reported in Table 3. The neat PLA e-fibers were degraded in a single degradation process, exhibiting a maximum temperature of 340 °C, which is in accordance with previously reported results [53]. Regarding the different electrospun PLA-based samples, only the addition of CNC increased the thermal stability of the e-fibers, exhibiting a maximum temperature of 364 °C, which is more than 20 °C above the T_m_ of the neat PLA e-fibers. This phenomenon can be attributed to the good interaction between the NPs and the matrix in which they are dispersed, as previously reported in literature. Sessini et al. reported an increase of 11 °C compared to the T_m_ of the neat PLA films [25]. Moreover, Arrieta et al. reported an increase in the T_m_ value of PLA-based e-fibers by 18 °C with 1 wt% CNC [54]. However, a shift of T_max_ to lower temperature values was observed in the other PLA-based e-fibers. That is, a decrease of 14 °C was observed for PLA/chitosan, which was the minimum among all of the samples studied. As described above, this sample presented the worst thermal stability, as also observed in previously reported results when reinforcing PLA films with chitosan-based NPs [55].

**Figure 2:**
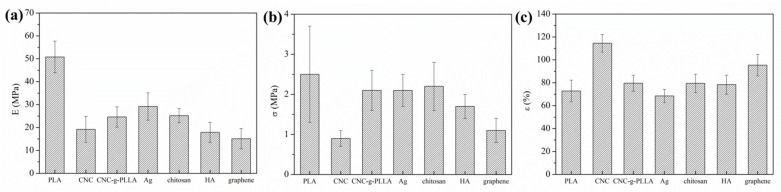
Mechanical response for the neat PLA and the reinforced PLA-based e-fibers. Elastic modulus (**a**), tensile strength (**b**), and elongation at break (**c**) for all of the samples studied.

To study the mechanical response of the PLA-based e-fibers, tensile tests were performed in order to obtain the elastic modulus €, tensile strength (σ), and elongation at break (ε at break). The results obtained are plotted in Figure 2 and summarized in Table 4.

Firstly, all of the PLA-based e-fibers showed statistically significantly different values of elastic modulus in comparison with neat PLA e-fibers (*p* < 0.05). In fact, neat PLA e-fibers showed an E of 50.8 ± 6.9 MPa, which is in agreement with previously reported results [8]. This value is the maximum value observed for any of the studied PLA-based electrospun nanocomposite mats. Both organic and inorganic NPs clearly decrease the elastic modulus of the electrospun mats, regardless of the nature of the different NPs. This behavior is in agreement with the plasticizing effect previously reported in literature. In this respect, Cacciotti et al. studied the mechanical properties of PLA/Ag and PLA/CNC electrospun fibers and reported a decrease in E values of 11% and 67%, respectively [56]. However, this behavior is the opposite of that studied previously in PCL matrix, where the same organic and inorganic NPs enhanced the elastic modulus in all of the PCL-based e-fibers [24].

Furthermore, regarding tensile strength, PLA-based electrospun nanocomposites can be divided into two different groups on the basis of the ANOVA: those where the tensile strength does not change statistically significantly in comparison to neat PLA, and those where this property decreases. CNC, CNC-g-PLLA and chitosan, i.e., the organic NPs used, showed tensile strength values of 0.9 ± 0.2 MPa, 1.1 ± 0.3 MPa and 1.7 ± 0.3 MPa, respectively, which are values lower than that of 2.5 ± 1.2 presented by the neat PLA e-fibers, confirming their plasticizing effect.

On the other hand, the inorganic NPs, HA and Ag, and graphene did not change the tensile strength in a statistically significant manner, with values of 2.2 ± 0.6 MPa and 2.1 ± 0.4 MPa for PLA/Ag and PLA/HA being reported, respectively. This behavior is in agreement with previous results reported on PLA electrospun fibers reinforced with different NPs. Kotrotsos et al. reported similar mechanical properties for neat PLA and PLA reinforced with HA 1% *w*/*w* with respect to tensile strength, with values of 3.5 MPa and 4.5 MPa, respectively [57].

However, as summarized in Table 4, the elongation at break was successfully improved in almost all of the PLA-based e-fibers. Both organic and inorganic NPs result in improvements to this mechanical property, regardless of their chemical nature. Moreover, a statistically significant difference was observed in the PLA/CNC and PLA/CNC-g-PLLA samples. CNC and CNC-g-PLLA NPs clearly increased the elongation at break of the neat PLA e-fibers to 114.5 ± 7.7%, the highest value reported. This fact underlines the ability of these systems to improve the stiffness of neat PLA, which is in contrast to its typical intrinsic fragility. An increment of about 30% in the elongation at break of PLA/CNC-g-PLLA e-fibers with respect to PLA e-fibers suggests a plasticizing effect of CNC on the PLA matrix, as well as the better dispersion achieved as a result of grafting PLLA chains onto the CNC surfaces. The plasticizer phenomenon can also explain the decrease in the elastic modulus of the PLA-based e-fibers with respect to the neat ones, while the increase in the degree of crystallinity was in good agreement with the good dispersion of the NPs inside the PLA matrix. In our previous work, a similar behavior was reported when grafting PCL chains onto the CNC surface for use in PCL-based e-fibers, evidencing that the “*grafting from*” reaction can be carried out to improve the dispersion of CNC through polymeric matrices [24].

**Figure 3:**
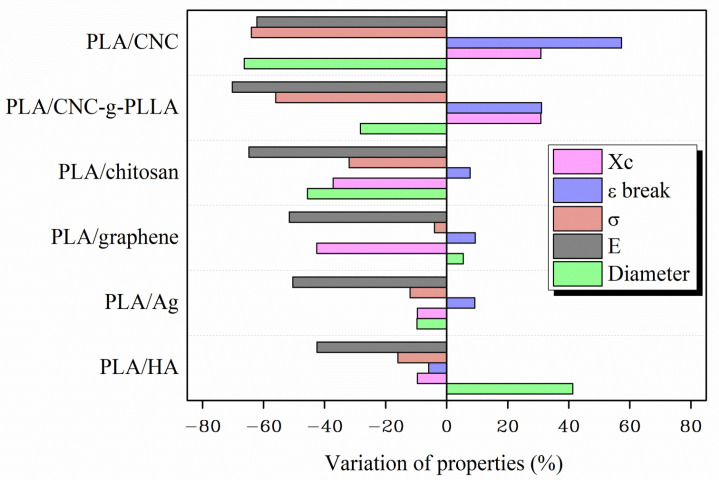
Variation of the properties of the woven non-woven electrospun nanocomposites systems with respect to the PLA values.

Finally, for a global understanding of the morphology and the thermal and mechanical behavior of all of the PLA-based e-fibers, the variation of their properties with respect to those of neat PLA are plotted in Figure 3. In this way, a better visualization of the effect of the addition of different organic and inorganic NPs on the main properties of the electrospun nanocomposites can be obtained. T_g_ and T_m_ were not plotted, as no detectable differences were found between the different systems.

It is quite difficult to produce a general classification of the studied PLA-based e-fibers in terms of the addition of organic or inorganic NPs. First of all, the average diameter of the e-fibers was clearly affected by the addition of different NPs. While CNC, CNC-g-PLLA, Ag and chitosan NPs significantly decreased the average diameter, the addition of HA NPs increased the average diameter values by up to 41% with respect to the neat PLA e-fibers. On the contrary, the addition of CNC and CNC-g-PLLA increased the degree of crystallinity of the nanocomposite e-fibers even if their average diameters were smaller than those of the other systems studied. From a mechanical point of view, a general classification can be performed. While the elastic modulus and tensile strength decreased in all of the nanocomposite e-fibers, the elongation at break clearly increased in almost all of the samples. In particular, the CNC and CNC-g-PLLA e-fibers showed the highest values of elongation at break, 114.5 ± 7.7% and 95.4 ± 9.4%, respectively, representing increments of 57% and 31% in comparison with neat PLA e-fibers. Therefore, we conclude that it is not possible to find clear differences between the addition of organic NPs and the addition of inorganic NPs with respect to the properties of PLA e-fibers. However, the addition of both CNC and CNC-g-PLLA induced the formation of e-fibers with smaller diameters, higher degrees of crystallinity, and more elastic fibers.

**Figure 4:**
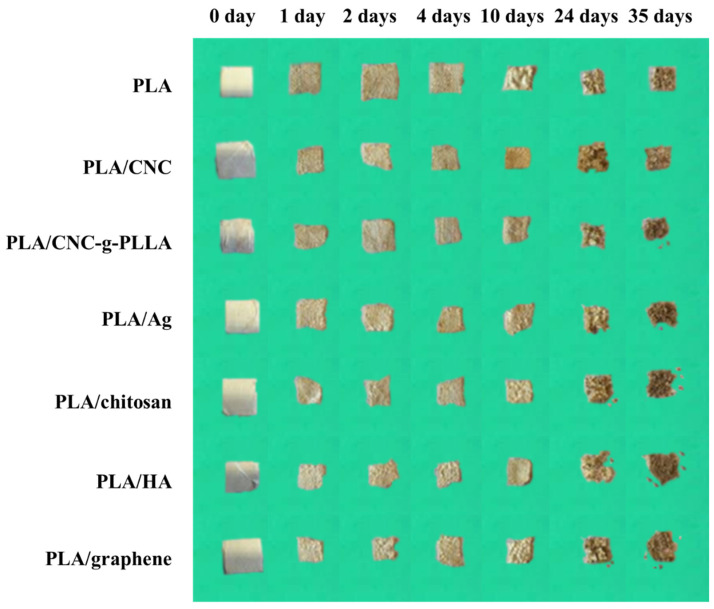
PLA-based e-fibers after composting degradation.

Additionally, a composting disintegration experiment was performed on the PLA-based e-fibers. In Figure 4, photographs for the electrospun nanocomposites degraded for different numbers of days under composting conditions are shown.

It is easy to note that all of the PLA-based e-fibers present evident signs of degradation starting from the first day, such as the slight color change and the increased superficial roughness. The increased opacity of the electrospun PLA-based mats was ascribed to the hydrolytic degradation process; water absorption, as along with the presence of products formed during the hydrolytic degradation process, change the refraction index of the materials [18]. Additionally, a reduction in the size of the samples was observed in all of the PLA-based e-fibers. This reduction started after 4 days under composting conditions in the neat PLA e-fibers, while in the reinforced samples, the reduction started after only one day. These findings could be related to the higher hydrophilic surface provided by the fillers, as was observed in previous works on PLA-based electrospun mats reinforced with organic fillers such as CNC [54]. Similarly, it has been shown that for inorganic nanoparticles such as silver nanoparticles [58] and hydroxyapatite [43], the presence of particles that providing a hydrophilic character to the surface of the materials favors the disintegration process. Thus, the addition of both the organic and inorganic NPs speeds up the disintegration phenomenon. During composting, the material disintegration process begins with a hydrolysis process; the hydrolytic degradation of PLA starts with water absorption followed by the breaking of the polymer chain via ester bonds in the amorphous phase [18,59], which is further attacked by the enzymes of microorganisms at the initial stage of this process. It should be highlighted that the electrospun PLA-based nanocomposites exhibited reduced crystallinity, with the exception of the PLA/CNC and PLA/CNC-*g*-PLLA electrospun nanocomposites (see Table 3). This reduced crystallinity increased the degradation rate of the electrospun nanocomposites, since the amorphous regions, with less ordered structures than the crystalline fractions, enabled the optimal action of microorganisms with respect to polymer disintegration [18,54]. In the case of the more crystalline nanocellulose reinforced materials, PLA/CNC and PLA/CNC-*g*-PLLA, the large number of hydroxyl groups on the nanocrystal surface provides the materials with a high level of hydrophilicity, thus accelerating the hydrolytic disintegration process, which in turn governs the disintegration rate of such electrospun nanocomposites. Once the hydrolytic disintegration process has started, the crystalline regions cannot delay the overall disintegration process.

Finally, after 35 days it was practically impossible to separate the materials from the compost medium, and all of the samples were visibly disintegrated. These results are in good agreement with the literature, wherein Arrieta et al. previously reported that all of the PLA-based mats disintegrated fully under composting conditions in less than 2 months. Specifically, the electrospun PLA-based mats became breakable into small pieces, and were almost fully disintegrated after 37 days [18].

## 3. Materials and Methods

Polylactic acid (PLA 3051D) was supplied by NatureWorks (NatureWorks LLC, Minnetonka, MN, USA) with 3% d-lactic acid monomer and a molecular weight (Mn) of 142 × 10^4^ g·mol^−1^. Chitosan (degree of deacetylation >75%) and microcrystalline cellulose, as well as commercial hydroxyapatite nanoparticles, HA NPs, with a particle size of about 30 nm, were purchased from Sigma-Aldrich (Madrid, Spain). Silver nanoparticles, Ag NPs, (P203, Cima Nano Tech, Saint Paul, MN, USA) were previously purified using a thermal treatment, obtaining specific surface area of 4.9 m^2^·g^−1^ and a particle size distribution ranging from 20 to 70 nm. Graphene nanoplatelets were supplied by Cheap Tube, Inc., Cambridgeport, VT, USA (Grade 2). As indicated, they possessed a surface area of about 100 m^2^·g^−1^, and an average thickness of a bit less than 10 nm. They were used as supplied by the manufacturer without any functionalization processes. Chloroform (99.6% purity), CF, and dimethylformamide (DMF) (99.5% purity) were supplied by Sigma Aldrich (Madrid, Spain).

Cellulose nanocrystals were obtained by sulfuric acid hydrolysis of 64% (*wt*/*wt*) microcrystalline cellulose, MCC, stirred at 45 °C for 30 min. The acid was further eliminated by centrifugation; the sediment was then dialyzed until a neutral pH was reached. An ion exchange resin was added to the cellulose suspension for 24 h and it was then removed by filtration followed by ultrasonic treatment. Cellulose nanocrystal, CNC, solutions were then neutralized (1.0% (*w*/*w*) of 0.25 mol·L^−1^ NaOH). Finally, the CNC solution was sonicated to obtain a stable NP suspension.

Chemical modification of the CNC surface was performed by grafting PLLA chains onto the CNC surface by means of ring opening polymerization (ROP) of l-Lactic acid (l-LA), using the surface hydroxyl groups of the CNC as the initiator, as schematically shown in the Supplementary Materials, Figure S4.

Briefly, the aqueous suspension of CNC was solvent-exchanged with acetone, then with dichloromethane and finally with previously dried toluene with phosphorus pentoxide. For each solvent exchange step, the solution was centrifuged and re-dispersed three times.

CNC and functionalized CNC were characterized on the basis of Raman spectra (inVia, Wotton-under-Edge, UK), using a Renishaw InVia Reflex Raman system. An optical microscope was coupled to the system. The Raman scattering was excited using a diode laser at a wavelength of 785 nm. The laser beam was focused on the sample using a 100 × 0.85 microscope objective. The laser power at the sample was 350 mW. The length of exposure was 10 s, and there were 10 accumulations for the Raman measurements. Fourier transform infrared, FT-IR spectra were obtained in the attenuated total reflectance ATR mode. The measurements were performed using a Spectrum One FTIR spectrometer Perkin Elmer (Perkin Elmer Spain, S.L., Madrid, Spain) equipped with a diamond internal reflection element in the range of 650–4000 cm^−1^ with a resolution of 1 cm^−1^ and 16 scans.

Electrospun fibers were prepared by means of a coaxial Electrospinner Yflow 2.2.D-XXX (Nanotechnology Solutions, Malaga, Spain) using a standard vertical configuration, equipped with two concentric needles, and connected to a high-voltage power supply. The polymer solution flowed through the inner needle and the solvent flowed through the outer one.

The PLA electrospun fibers were prepared from polymer solutions of PLA 8 wt% in a solvent mixture of CHCl_3_:DMF (4:1) using magnetic stirring for 24 h at room temperature. The same solution was used to obtain PLA-based e-fibers by adding 1 wt% with respect to the polymer matrix of organic and inorganic NPs, including CNC, CNC-g-PLA, Ag, chitosan, HA, and graphene.

To prepare electrospun nanocomposite fibers, NPs were dispersed separately (1 wt%) in the same mix of solvents using magnetic stirring for 2 h and then were sonicated for 2 min. Finally, to achieve homogeneous dispersion of the NPs in the dissolved matrix, the polymer solution and NP suspensions were mixed, stirred (2 h) and sonicated (2 min) to form solutions with 1 wt% with respect to the matrix in the final composition.

The polymer solutions flowed through the inner needle, and the same mixture of solvent used for the polymer solutions flowed through the outer needle. The applied positive and negative voltages were set at 10 and −10 kV, respectively. The polymer flow rate and the solvent flow rate were both fixed at 0.1 mL·h^−1^. Electrospun mats were randomly collected in a grounded aluminum foil collector situated perpendicular to the charged spinneret at a distance of 15 cm. The obtained electrospun mats were vacuumed for 48 h in a vacuum chamber to eliminate residual solvents before testing.

The morphology of the PLA-based e-fibers was analyzed by means of scanning electron microscopy (SEM). To this end, the electrospun fibers were sputtered with a gold/palladium layer and observed using a PHILIPS XL30 Scanning Electron Microscope (SEM, Phillips, Eindhoven, The Netherlands). The fiber diameters were statistically calculated by means of the Fib_thick software, executed using the image analysis platform Fiji based on ImageJ.

TGA analysis was performed to study the thermal degradation of the electrospun samples. Thermogravimetric analysis was performed in a TA-TGA Q500 thermal analyzer (TA Instruments, New Castle, DE, USA). Dynamic TGA experiments were performed under a nitrogen atmosphere (flow rate of 60 mL·min^−1^). Samples were heated from room temperature to 700 °C at 10 °C·min^−1^. In this case, the maximum degradation temperature (T_max_) was calculated from the first derivative of the TGA curve.

The thermal behavior and the degree of crystallinity, Xc, were studied by means of Differential Scanning Calorimetry (DSC). DSC experiments were conducted using a Mettler Toledo DSC822e instrument (Mettler-Toledo, Schwarzenbach, Switzerland) under a nitrogen atmosphere (50 mL·min^−1^). Sample weights of about 4 mg were sealed in aluminum pans and heated from −90 to 100 °C at a heating rate of 10 °C·min^−1^. The glass transition temperature (T_g_) was taken at the midpoint of the heat capacity changes. The melting temperature (T_m_) and cold crystallization temperature (T_cc_) were obtained from the first heating, and the degree of crystallinity (χ_c_) was calculated using Equation (1):

(1) $$\chi_{c}=100\%\times \left[\frac{\Delta H_{m}-\Delta H_{cc}}{\Delta H_{m}^{{}^{\circ}}}\right]$$

where Δ***H_m_*** is the melting enthalpy, Δ***H_cc_*** is the cold crystallization enthalpy, and $\Delta H_{m}^{{}^{\circ}}$ is the melting heat associated with pure crystalline PLA (93.6 J·g^−1^) [11].

The mechanical properties of the PLA-based electrospun nanocomposite mats were investigated on the basis of tensile test measurements performed at room temperature using an Instron dynamometer (model 3366) equipped with a 100 N load cell at a crosshead speed of 10 mm·min^−1^ and an initial length of 30 mm. Dog-bone samples were prepared with a width of 2 mm and a thickness of about 240 μm from the mats, and at least five specimens were tested for each formulation. On the basis of these experiments, the elastic modulus, taken as the slope of the curve between 0% and 2% deformation, the elongation at break, and the maximum strain reached were obtained. Specimens were statistically analyzed by one-way analysis of variance (ANOVA) using the statistical computer package Statgraphics Centurion XVII (Statpoint Technologies, Inc., Warrenton, VA, USA). To identify which groups were significantly different from other groups, mean comparisons were performed employing Tukey’s test with a 95% confidence level [13].

The biodegradation analysis under composting conditions was performed in accordance with the ISO 20200 standard [60]. Solid synthetic waste was prepared by mixing 5% compost inoculum with 15% rabbit food, 5% starch, 2.5% sugar, 1% urea, 1.5% corn oil and 20% sawdust. The water content of the substrate was around 50 wt%, and the aerobic conditions were ensured by mixing it softly. Samples (cut in 15 × 15 mm^2^) were weighed and buried at a depth of 4–6 cm in perforated plastic containing the prepared mix and incubated at 58 °C. Every day, the weight of the vessels was measured. The weight lost corresponds to the water evaporation, so they were re-filled with water in order to preserve the composting conditions. Each sample was recovered at different times of disintegration, cleaned with distilled water, and dried in an oven at 37 °C during 24 h.

## 4. Conclusions

Different organic (cellulose nanocrystals, chitosan and graphene) and inorganic (silver and hydroxyapatite) NPs were used as nanofillers in order to obtain PLA-based e-fibers. Specifically, cellulose nanocrystals were also functionalized by means of a “*grafting from*” reaction, grafting PLLA chains onto the CNC surface. After optimizing the electrospinning conditions for the neat PLA e-fibers, all of the different NPs were added to the polymer solution at 1 wt% with respect to the matrix and were successfully electrospun. All of the randomly oriented PLA-based e-fibers exhibited smooth and uniform morphologies with no beads, as well as average diameters in the micron range. All of the e-fibers exhibited good thermal stability, with values similar to those obtained for neat PLA. Moreover, the use of CNC and CNC-g-PLLA NPs increased the degree of crystallinity of the e-fibers. From a mechanical point of view, no increase was found in the elastic modulus or tensile strength of any of the PLA-based electrospun mats when using either organic or inorganic NPs. However, the elongation at break of the materials was successfully improved in almost all of the samples. In particular, CNC and CNC-g-PLLA NPs increased the elongation at break from the value of 72.8 ± 9.4% obtained for the neat PLA to 114.5 ± 7.7 and 95.4 ± 9.4%, respectively. Finally, degradation under composting conditions was carried out, revealing visible disintegration after 35 days for all samples.

## Figures and Tables

**Table 1:** Optimization of the electrospinning process.

Samples	C (% wt)	Q_s_ (mL/h)	Q_p_ (mL/h)	V+ (kV)	V− (kV)	Fibers Diameter (µm)
PLA_1_	1	0.3	2.0	6.3	−8.0	No fiber formation
PLA_2_	1	0.3	0.3	6.4	−0.5	No fiber formation
PLA_3_	1	0.1	0.1	10.0	−10.0	No fiber formation
PLA_4_	1	0.3	0.3	10.6	−10.5	No fiber formation
PLA_5_	1	0.8	0.8	10.6	−10.5	No fiber formation
PLA_6_	1	0.8	0.8	11.4	−1.4	No fiber formation
PLA_7_	2	0.3	2.0	6.3	−8.0	No fiber formation
PLA_8_	2	0.3	0.3	6.4	−0.5	No fiber formation
PLA_9_	2	0.1	0.1	10.0	−10.0	0.18 ± 0.01
PLA_10_	2	0.3	0.3	10.6	−10.5	No fiber formation
PLA_11_	2	0.8	0.8	10.6	−10.5	No fiber formation
PLA_12_	2	0.8	0.8	11.4	−1.4	No fiber formation
PLA_13_	4	0.3	2.0	6.3	−8.0	0.25 ± 0.01
PLA_14_	4	0.3	0.3	6.4	−0.5	No fiber formation
PLA_15_	4	0.1	0.1	10.0	−10.0	0.36 ± 0.03
PLA_16_	4	0.3	0.3	10.6	−10.5	0.33 ± 0.02
PLA_17_	4	0.8	0.8	10.6	−10.5	0.33 ± 0.02
PLA_18_	4	0.8	0.8	11.4	−1.4	No fiber formation
PLA_19_	5	0.3	2.0	6.3	−8.0	0.31 ± 0.02
PLA_20_	5	0.3	0.3	6.4	−0.5	No fiber formation
PLA_21_	5	0.1	0.1	10.0	−10.0	0.76 ± 0.05
PLA_22_	5	0.1	0.0	10.0	−10.0	1.16 ± 0.10
PLA_23_	5	1.0	0.1	10.0	−10.0	0.70 ± 0.06
PLA_24_	5	1.0	1.0	10.0	−10.0	0.76 ± 0.04
PLA_25_	5	0.8	0.8	10.0	−10.0	0.75 ± 0.06
PLA_26_	5	0.1	0.3	10.0	−10.0	0.43 ± 0.04
PLA_27_	5	0.1	0.5	10.0	−10.0	0.61 ± 0.03
PLA_28_	5	0.1	0.7	10.0	−10.0	0.34 ± 0.02
PLA_29_	5	0.1	1.0	10.0	−10.0	0.37 ± 0.02
PLA_30_	5	0.3	0.3	10.6	−10.5	0.40 ± 0.03
PLA_31_	5	0.8	0.8	10.6	−10.5	0.32 ± 0.02
PLA_32_	5	0.8	0.8	11.4	−1.4	No fiber formation
PLA_33_	6	0.3	2.0	6.3	−8.0	0.80 ± 0.05
PLA_34_	6	0.3	0.3	6.4	−0.5	0.51 ± 0.03
PLA_35_	6	0.1	0.1	10.0	−10.0	0.83 ± 0.07
PLA_36_	6	0.3	0.3	10.6	−10.5	0.73 ± 0.05
PLA_37_	6	0.8	0.8	10.6	−10.5	0.68 ± 0.05
PLA_38_	6	0.8	0.8	11.4	−1.4	0.68 ± 0.06
PLA_39_	7	0.3	2.0	6.3	−8.0	0.80 ± 0.05
PLA_40_	7	0.3	0.3	6.4	−0.5	0.88 ± 0.06
PLA_41_	7	0.1	0.1	10.0	−10.0	0.91 ± 0.07
PLA_42_	7	0.3	0.3	10.6	−10.5	0.83 ± 0.06
PLA_43_	7	0.8	0.8	10.6	−10.5	0.85 ± 0.05
PLA_44_	7	0.8	0.8	11.4	−1.4	0.82 ± 0.03
PLA_45_	8	0.3	2.0	6.3	−8.0	1.12 ± 0.09
PLA_46_	8	1.0	1.0	10.0	−10.0	0.92 ± 0.10
PLA_47_	8	0.3	0.3	10.6	−10.5	0.82 ± 0.09
PLA_48_	8	0.8	0.8	10.6	−10.5	1.22 ± 0.10
PLA_49_	8	0.8	0.8	11.4	−1.4	0.99 ± 0.10
PLA_50_	10	0.3	2.0	6.3	−8.0	2.03 ± 0.22

**Table 2:** Diameter of PLA-based e-fibers.

Samples	Diameter (µm)
PLA	0.92 ± 0.10 ^a^
PLA/CNC	0.31 ± 0.03 ^d^
PLA/CNC-g-PLLA	0.66 ± 0.05 ^b,c^
PLA/Ag	0.83 ± 0.08 ^a,b^
PLA/chitosan	0.50 ± 0.04 ^c,d^
PLA/HA	1.30 ± 0.10 ^e^
PLA/graphene	0.97 ± 0.07 ^a^
F ratio	61.92
*p*-Value	0.0000 *

Different letters in the column indicate significant differences according to Tukey’s test (*p* < 0.05). * Values are significant at *p* < 0.05.

**Table 3:** Thermal characterization of the neat PLA and the reinforced PLA-based e-fibers.

Samples	T_g_ (°C)	T_m_ (°C)	X_c_ (%)	T_max_ (°C)
PLA	55	152	9.4	340
PLA/CNC	55	150	12.3	364
PLA/CNC-g-PLLA	54	151	12.3	335
PLA/Ag	56	150	8.5	334
PLA/chitosan	60	151	5.4	326
PLA/HA	56	149	8.5	329
PLA/graphene	55	151	5.9	340

**Table 4:** Mechanical properties for neat PLA and reinforced PLA-based e-fibers.

Samples	E (MPa)	σ (MPa)	ε Break (%)
PLA	50.8 ± 6.9 ^a^	2.5 ± 1.2 ^a^	72.8 ± 9.4 ^a^
PLA/CNC	19.2 ± 5.7 ^b,c^	0.9 ± 0.2 ^b^	114.5 ± 7.7 ^b^
PLA/CNC-g-PLLA	15.1 ± 4.4 ^b^	1.1 ± 0.3 ^b^	95.4 ± 9.4 ^c^
PLA/Ag	25.2 ± 3.1 ^c,d^	2.2 ± 0.6 ^a^	79.5 ± 8.0 ^a^
PLA/chitosan	17.9 ± 4.4 ^b,c^	1.7 ± 0.3 ^a,b^	78.4 ± 8.3 ^a^
PLA/HA	29.2 ± 6.0 ^d^	2.1 ± 0.4 ^a^	68.5 ± 5.8 ^a^
PLA/graphene	24.6 ± 4.4 ^c,d^	2.4 ± 0.5 ^a^	79.6 ± 7.0 ^a^
F ratio	22.33	9.80	27.10
*p*-Value	0.0000 *	0.0000 *	0.0000 *

Different letters in the column indicate statistically significant differences according to Tukey’s test (*p* < 0.05). * Values are significant at *p* < 0.05.

## Data Availability

Not applicable.

## Supplementary Materials

The following are available online. Figure S1: Vibrational spectroscopy: (a) FTIR spectra for PLA, CNC and CNC-g-PLLA, (b) Raman spectra for PLA, CNC and CNC-g-PLLA, and (c) thermogravimetric analysis of CNC-g-PLLA with grafted PLA, Figure S2: SEM images and average dimension of: (a) CNC, (b) CNCg-PLLA, (c) Ag, (d) chitosan, (e) HA and (f) graphene nanoparticles, Figure S3: SEM image of neat PLA e-fibers, Figure S4: CNC surface chemical modification by grafting PLLA chains onto the CNC surface by ring-opening polymerization (ROP).

## Sample Availability

Samples of the compounds are not available from the authors.

## References

[1] Raquez J.M., Habibi Y., Murariu M., Dubois P. (2013). Polylactide (PLA)-based nanocomposites. Prog. Polym. Sci..

[2] Maraveas C. (2020). The Sustainability of Plastic Nets in Agriculture. Sustainability.

[3] Arrieta M., Samper M., Aldas M., López J. (2017). On the Use of PLA-PHB Blends for Sustainable Food Packaging Applications. Materials.

[4] Tyler B., Gullotti D., Mangraviti A., Utsuki T., Brem H. (2016). Polylactic acid (PLA) controlled delivery carriers for biomedical applications. Adv. Drug Deliv. Rev..

[5] Islam G.M.N., Collie S., Qasim M., Ali M.A. (2020). Highly Stretchable and Flexible Melt Spun Thermoplastic Conductive Yarns for Smart Textiles. Nanomaterials.

[6] Leonés A., Peponi L., Lieblich M., Benavente R., Fiori S. (2020). In vitro degradation of plasticized PLA electrospun fiber mats: Morphological, thermal and crystalline evolution. Polymers.

[7] Farah S., Anderson D.G., Langer R. (2016). Physical and mechanical properties of PLA, and their functions in widespread applications—A comprehensive review. Adv. Drug Deliv. Rev..

[8] Leonés A., Sonseca A., López D., Fiori S., Peponi L. (2019). Shape memory effect on electrospun PLA-based fibers tailoring their thermal response. Eur. Polym. J..

[9] Mujica-Garcia A., Hooshmand S., Skrifvars M., Kenny J.M., Oksman K., Peponi L. (2016). Poly(lactic acid) melt-spun fibers reinforced with functionalized cellulose nanocrystals. RSC Adv..

[10] Sonseca A., Madani S., Muñoz-Bonilla A., Fernández-García M., Peponi L., Leonés A., Rodríguez G., Echeverría C., López D. (2020). Biodegradable and Antimicrobial PLA–OLA Blends Containing Chitosan-Mediated Silver Nanoparticles with Shape Memory Properties for Potential Medical Applications. Nanomaterials.

[11] Peponi L., Navarro-Baena I., Báez J.E., Kenny J.M., Marcos-Fernández A. (2012). Effect of the molecular weight on the crystallinity of PCL-b-PLLA di-block copolymers. Polymer.

[12] Navarro-Baena I., Kenny J.M., Peponi L. (2014). Crystallization and thermal characterization of biodegradable tri-block copolymers and poly(ester-urethane)s based on PCL and PLLA. Polym. Degrad. Stab..

[13] Arrieta M.P., Gil A.L., Yusef M., Kenny J.M., Peponi L. (2020). Electrospinning of PCL-based blends: Processing optimization for their scalable production. Materials.

[14] Mujica-Garcia A., Navarro-Baena I., Kenny J.M., Peponi L. (2014). Influence of the Processing Parameters on the Electrospinning of Biopolymeric Fibers. J. Renew. Mater..

[15] Khorshidi S., Solouk A., Mirzadeh H., Mazinani S., Lagaron J.M., Sharifi S., Ramakrishna S. (2016). A review of key challenges of electrospun scaffolds for tissue-engineering applications. J. Tissue Eng. Regen. Med..

[16] Toncheva A., Spasova M., Paneva D., Manolova N., Rashkov I. (2014). Polylactide (PLA)-Based Electrospun Fibrous Materials Containing Ionic Drugs as Wound Dressing Materials: A Review. Int. J. Polym. Mater. Polym. Biomater..

[17] Müller K., Bugnicourt E., Latorre M., Jorda M., Echegoyen Sanz Y., Lagaron J., Miesbauer O., Bianchin A., Hankin S., Bölz U. (2017). Review on the Processing and Properties of Polymer Nanocomposites and Nanocoatings and Their Applications in the Packaging, Automotive and Solar Energy Fields. Nanomaterials.

[18] Arrieta M.P., Perdiguero M., Fiori S., Kenny J.M., Peponi L. (2020). Biodegradable electrospun PLA-PHB fibers plasticized with oligomeric lactic acid. Polym. Degrad. Stab..

[19] Leonés A., Lieblich M., Benavente R., Gonzalez J.L., Peponi L. (2020). Potential applications of magnesium-based polymeric nanocomposites obtained by electrospinning technique. Nanomaterials.

[20] Habibi Y., Lucia L.A., Rojas O.J. (2010). Cellulose nanocrystals: Chemistry, self-assembly, and applications. Chem. Rev..

[21] Vatansever E., Arslan D., Nofar M. (2019). Polylactide cellulose-based nanocomposites. Int. J. Biol. Macromol..

[22] Li T., Chen C., Brozena A.H., Zhu J.Y., Xu L., Driemeier C., Dai J., Rojas O.J., Isogai A., Wågberg L. (2021). Developing fibrillated cellulose as a sustainable technological material. Nature.

[23] Tavakolian M., Jafari S.M., van de Ven T.G.M. (2020). A Review on Surface-Functionalized Cellulosic Nanostructures as Biocompatible Antibacterial Materials. Nano-Micro Lett..

[24] Leonés A., Garcia A.M., Arrieta M.P., Salaris V., Lopez D., Kenny J.M., Peponi L. (2020). Organic and Inorganic PCL—Based Electrospun Fibers. Polymers.

[25] Sessini V., Navarro-Baena I., Arrieta M.P., Dominici F., López D., Torre L., Kenny J.M., Dubois P., Raquez J.M., Peponi L. (2018). Effect of the addition of polyester-grafted-cellulose nanocrystals on the shape memory properties of biodegradable PLA/PCL nanocomposites. Polym. Degrad. Stab..

[26] Zhou J., Li H., Li Y., Li X. (2021). V-Shaped amphiphilic polymer brushes grafted on cellulose nanocrystals: Synthesis, characterization and properties. J. Phys. Chem. Solids.

[27] Sivanesan I., Muthu M., Gopal J., Hasan N., Kashif Ali S., Shin J., Oh J.-W. (2021). Nanochitosan: Commemorating the Metamorphosis of an ExoSkeletal Waste to a Versatile Nutraceutical. Nanomaterials.

[28] Pillai C.K.S., Paul W., Sharma C.P. (2009). Chitin and chitosan polymers: Chemistry, solubility and fiber formation. Prog. Polym. Sci..

[29] Castro Marín A., Colangelo D., Lambri M., Riponi C., Chinnici F. (2020). Relevance and perspectives of the use of chitosan in winemaking: A review. Crit. Rev. Food Sci. Nutr..

[30] Yang H., Zhang Y., Zhou F., Guo J., Tang J., Han Y., Li Z., Fu C. (2020). Preparation, Bioactivities and Applications in Food Industry of Chitosan-Based Maillard Products: A Review. Molecules.

[31] Devlieghere F., Vermeulen A., Debevere J. (2004). Chitosan: Antimicrobial activity, interactions with food components and applicability as a coating on fruit and vegetables. Food Microbiol..

[32] Su W., Yu S., Wu D., Xia M., Wen Z., Yao Z., Tang J., Wu W. (2019). A critical review of cast-off crab shell recycling from the perspective of functional and versatile biomaterials. Environ. Sci. Pollut. Res..

[33] Martău G.A., Mihai M., Vodnar D.C. (2019). The Use of Chitosan, Alginate, and Pectin in the Biomedical and Food Sector—Biocompatibility, Bioadhesiveness, and Biodegradability. Polymers.

[34] Bayer I.S. (2017). Thermomechanical properties of polylactic acid-graphene composites: A state-of-the-art review for biomedical applications. Materials.

[35] Sun X., Huang C., Wang L., Liang L., Cheng Y., Fei W., Li Y. (2021). Recent Progress in Graphene/Polymer Nanocomposites. Adv. Mater..

[36] Geim A.K., Novoselov K.S. (2007). The rise of graphene. Nat. Mater..

[37] Peponi L., Tercjak A., Verdejo R., Lopez-Manchado M.A., Mondragon I., Kenny J.M. (2009). Confinement of functionalized graphene sheets by triblock copolymers. J. Phys. Chem. C.

[38] Verdejo R., Barroso-Bujans F., Rodriguez-Perez M.A., De Saja J.A., Lopez-Manchado M.A. (2008). Functionalized graphene sheet filled silicone foam nanocomposites. J. Mater. Chem..

[39] Su Z., Ding J., Wei G. (2014). Electrospinning: A facile technique for fabricating polymeric nanofibers doped with carbon nanotubes and metallic nanoparticles for sensor applications. RSC Adv..

[40] Safari B., Aghanejad A., Roshangar L., Davaran S. (2021). Osteogenic effects of the bioactive small molecules and minerals in the scaffold-based bone tissue engineering. Colloids Surf. B Biointerfaces.

[41] Zhou H., Lee J. (2011). Nanoscale hydroxyapatite particles for bone tissue engineering. Acta Biomater..

[42] Ribeiro Neto W.A., Pereira I.H.L., Ayres E., De Paula A.C.C., Averous L., Góes A.M., Oréfice R.L., Suman Bretas R.E. (2012). Influence of the microstructure and mechanical strength of nanofibers of biodegradable polymers with hydroxyapatite in stem cells growth. Electrospinning, characterization and cell viability. Polym. Degrad. Stab..

[43] Peponi L., Sessini V., Arrieta M.P., Navarro-Baena I., Sonseca A., Dominici F., Gimenez E., Torre L., Tercjak A., López D. (2018). Thermally-activated shape memory effect on biodegradable nanocomposites based on PLA/PCL blend reinforced with hydroxyapatite. Polym. Degrad. Stab..

[44] Sonseca A., Peponi L., Sahuquillo O., Kenny J.M., Giménez E. (2012). Electrospinning of biodegradable polylactide/hydroxyapatite nanofibers: Study on the morphology, crystallinity structure and thermal stability. Polym. Degrad. Stab..

[45] Venkatram M., Narasimha Murthy H.N.R., Gaikwad A., Mankunipoyil S.A., Ramakrishna S., Ayalasomayajula Ratna P. (2018). Antibacterial and Flame Retardant Properties of Ag-MgO/Nylon 6 Electrospun Nanofibers for Protective Applications. Cloth. Text. Res. J..

[46] Carbone M., Donia D.T., Sabbatella G., Antiochia R. (2016). Silver nanoparticles in polymeric matrices for fresh food packaging. J. King Saud Univ. Sci..

[47] Mikelonis A.M., Lawler D.F., Passalacqua P. (2016). Multilevel modeling of retention and disinfection efficacy of silver nanoparticles on ceramic water filters. Sci. Total Environ..

[48] Sotiriou G.A., Pratsinis S.E. (2010). Antibacterial activity by nanosilver ions and particles. AIChE Annu. Meet. Conf. Proc..

[49] Kim J.S., Kuk E., Yu K.N., Kim J.-H., Park S.J., Lee H.J., Kim S.H., Park Y.K., Park Y.H., Hwang C.-Y. (2007). Antimicrobial effects of silver nanoparticles. Nanomed. Nanotechnol. Biol. Med..

[50] Reneker D.H., Yarin A.L. (2008). Electrospinning jets and polymer nanofibers. Polymer.

[51] Liu C., Shen J., Liao C.Z., Yeung K.W.K., Tjong S.C. (2018). Novel electrospun polyvinylidene fluoride-graphene oxide-silver nanocomposite membranes with protein and bacterial antifouling characteristics. Express Polym. Lett..

[52] Au H.T., Pham L.N., Vu T.H.T., Park J.S. (2012). Fabrication of an antibacterial non-woven mat of a poly(lactic acid)/chitosan blend by electrospinning. Macromol. Res..

[53] Fontes M.R.V., da Rosa M.P., Fonseca L.M., Beck P.H., da Rosa Zavareze E., Dias A.R.G. (2021). Thermal stability, hydrophobicity and antioxidant potential of ultrafine poly (lactic acid)/rice husk lignin fibers. Braz. J. Chem. Eng..

[54] Arrieta M.P., López J., López D., Kenny J.M., Peponi L. (2016). Biodegradable electrospun bionanocomposite fibers based on plasticized PLA–PHB blends reinforced with cellulose nanocrystals. Ind. Crops Prod..

[55] Sonseca A., Madani S., Rodríguez G., Hevilla V., Echeverría C., Fernández-García M., Muñoz-Bonilla A., Charef N., López D. (2019). Multifunctional PLA Blends Containing Chitosan Mediated Silver Nanoparticles: Thermal, Mechanical, Antibacterial, and Degradation Properties. Nanomaterials.

[56] Cacciotti I., Fortunati E., Puglia D., Kenny J.M., Nanni F. (2014). Effect of silver nanoparticles and cellulose nanocrystals on electrospun poly(lactic) acid mats: Morphology, thermal properties and mechanical behavior. Carbohydr. Polym..

[57] Kotrotsos A., Yiallouros P., Kostopoulos V. (2020). Fabrication and characterization of polylactic acid electrospun scaffolds modified with multi-walled carbon nanotubes and hydroxyapatite nanoparticles. Biomimetics.

[58] Ramos M., Fortunati E., Peltzer M., Jimenez A., Kenny J.M., Garrigós M.C. (2016). Characterization and disintegrability under composting conditions of PLA-based nanocomposite films with thymol and silver nanoparticles. Polym. Degrad. Stab..

[59] Iglesias-Montes M.L., Luzi F., Dominici F., Torre L., Manfredi L.B., Cyras V.P., Puglia D. (2021). Migration and degradation in composting environment of active polylactic acid bilayer nanocomposites films: Combined role of umbelliferone, lignin and cellulose nanostructures. Polymers.

[60] UNE-EN-ISO (2015). Determination of the Degree of Disintegration of Plastic Materials under Simulated Composting Conditions in a Laboratory-scale Test.

