# Supplementary material for: PLA Electrospun Fibers Reinforced with Organic and Inorganic Nanoparticles: A Comparative Study
Source: Molecules. 2021 Aug 14;26(16):4925. doi: 10.3390/molecules26164925 (PMC8401602; doi:10.3390/molecules26164925)
Supplement: Supplementary file 1 [file molecules-26-04925-s001.zip › molecules-1301395-supplementary.pdf]

molecules

Article

PLA electrospun fibers reinforced with organic and inorganic nanoparticles: a comparative study

Adrián Leonés[1,2], Valentina Salaris[1], Alicia Mujica-Garcia[1,3], Marina P. Arrieta[1,4,5], Daniel Lopez[1,2], Marcela Lieblich[6], José Maria Kenny[1,3,*] and Laura Peponi[1,2,*]

1 Instituto de Ciencia y Tecnología de Polímeros (ICTP-CSIC), C/Juan de la Cierva 3, 28006 Madrid, Spain; aleones@ictp.csic.es (A.L.), v.salaris@ictp.csic.es (V.S.), alicia.mujica@unipg.it (A.M.-G), m.arrieta@upm.es (M.P.A), daniel.l.g@csic.es (D.L.), jose.kenny@unipg.it (J.M.K.), lpeponi@ictp.csic.es (L.P.)
2 Interdisciplinary Platform for Sustainable Plastics towards a Circular Economy−The Spanish National Research Council (SusPlast-CSIC), 28006 Madrid, Spain
3 Civil and Environmental Engineering Department and UDR INSTM, University of Perugia, Strada di Pentima 4, 05100 Terni, Italy
4 Departamento de Ingeniería Química Industrial y del Medio Ambiente, Escuela Politécnica Superior de Ingenieros Industriales, Universidad Politécnica de Madrid (ETSII-UPM), Calle José Gutiérrez Abascal 2, 28006, Madrid, Spain
5 Grupo de Investigación: Polímeros, Caracterización y Aplicaciones (POLCA), 28006 Madrid, Spain.
6 Centro Nacional de Investigaciones Metalúrgicas (CENIM-CSIC), Spain; marcela@cenim.csic.es (M.L.)
* Correspondence: lpeponi@ictp.csic.es; jose.kenny@unipg.it

molecules

MDPI

Supporting Information

Vibrational spectroscopy study was conducted in order to compare the neat PLA, CNC and to observe the presence of PLLA chains in CNC-g-PLLA. As can be seen in Figure S1, FTIR spectrum of neat PLA and CNC are reported where the characteristics peaks can be observed. In the CNC spectrum (Figure S1a), a broad peak in the region of 3200 cm^{-1} to 3600 cm^{-1} can be observed corresponding to hydroxyl groups while in PLA spectrum the peak related to carboxyl group can be observed in 1750 cm^{-1}. These two main peaks can be observed in CNC-g-PLLA spectrum confirming the presence of PLLA chains onto CNC surface. In addition, Raman spectroscopy analysis was carried out and reported in Figure S1b. As can be observed, the characteristics peaks of both, PLA and CNC, are present in the CNC-g-PLLA Raman spectrum confirming the success of the grafting reaction.

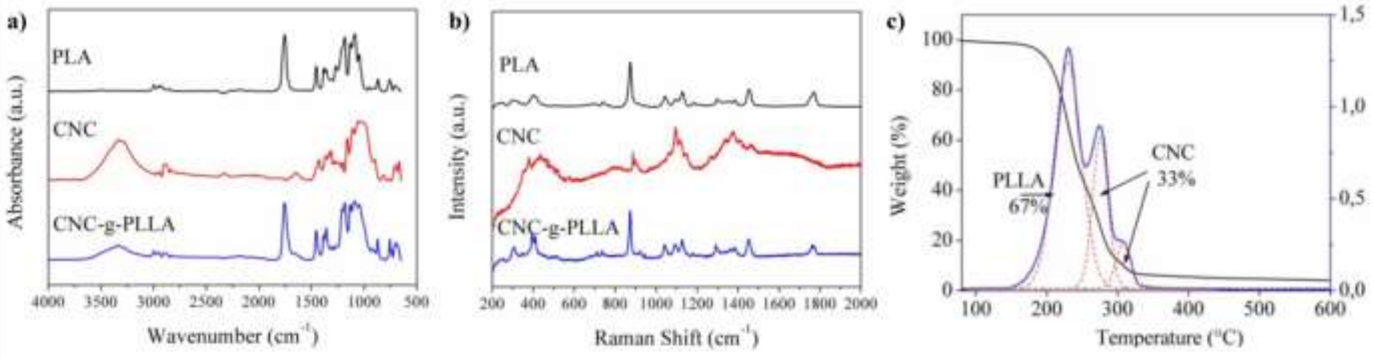

Figure S1. Vibrational spectroscopy: (a) FTIR spectra for PLA, CNC and CNC-g-PLLA, (b) Raman spectra for PLA, CNC and CNC-g-PLLA, and (c) thermogravimetric analysis of CNC-g-PLLA with grafted PLA chains**.**

Thermogravimetric analysis is also carried out in order to determinate the relative percentage of CNC and PLLA in CNC-g-PLLA nanoparticles. Two peaks can be observed in the derivate of weight loss curve reported in Figure S1c, one corresponding to the thermal degradation of PLLA close to 230 °C and one corresponding to the thermal degradation of CNC close to 275 °C. The amount of the components was calculated by fitting the curves with two Gaussian curves resulting in 67 wt% and 33 wt% PLLA chains and amount of CNC, respectively.

However, previously to the optimization of the electrospinning process, the morphological analysis of both organic and inorganic NPs was carried out. In Figure S2, the images of the different NPs as well as their average dimension are reported. In particular, CNC and CNC-g-PLLA showed rod-like morphology, with average length of 179 ± 20 nm and 298 ± 56 nm, respectively. Grafting PLLA chains onto CNC surface increases both the average length and diameters of CNC-g-PLLA NPs up to 40 % with respect neat CNC increasing their values from 14.1 ± 1.6 nm for neat CNC to 17.7 ± 2.9 nm for CNC-g-PLLA.

molecules

MDPI

Regarding the morphology of the commercial NPs, Ag, chitosan and HA, all of them showed spherical morphology with averages diameter values smaller than 30 nm, specifically 22.4 ± 2.4 nm, 23.7 ± 2.3 nm and 18.8 ± 2.0 nm, respectively, while graphene nanoplatelets show a length of about 290 nm.

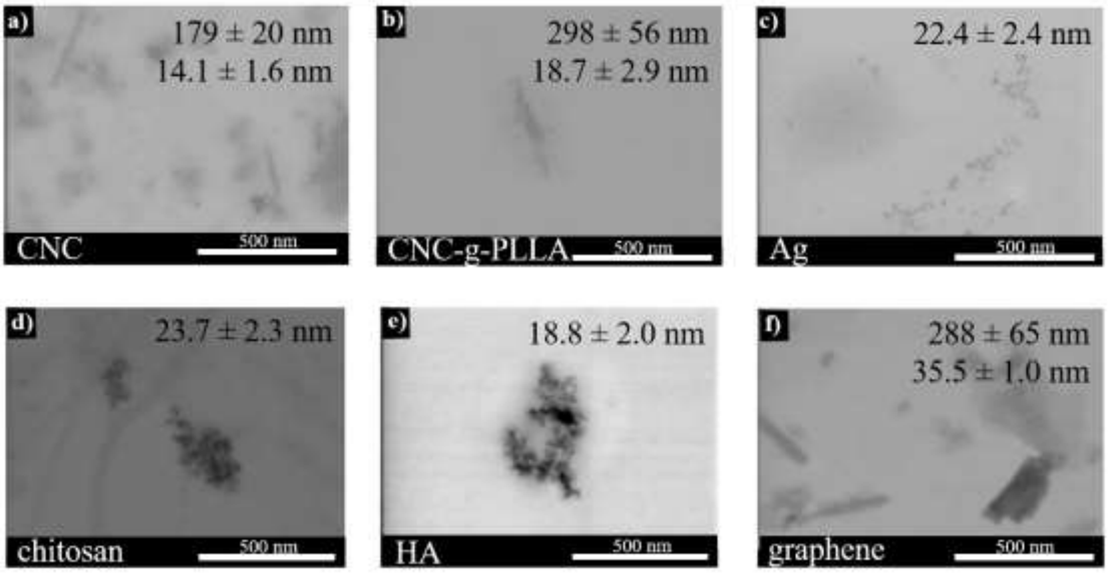

Figure S2. SEM images and average dimension of: (a) CNC, (b) CNC-g-PLLA, (c) Ag, (d) chitosan, (e) HA and (f) graphene nanoparticles.

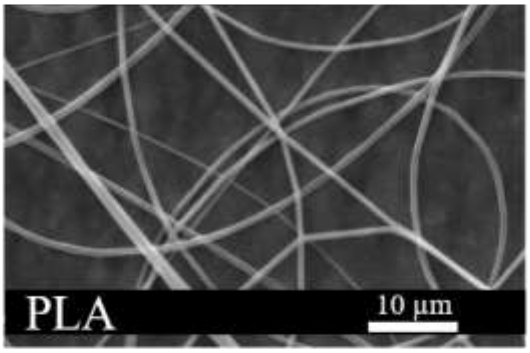

Figure S3. SEM image of neat PLA e-fibers.

molecules

MDPI

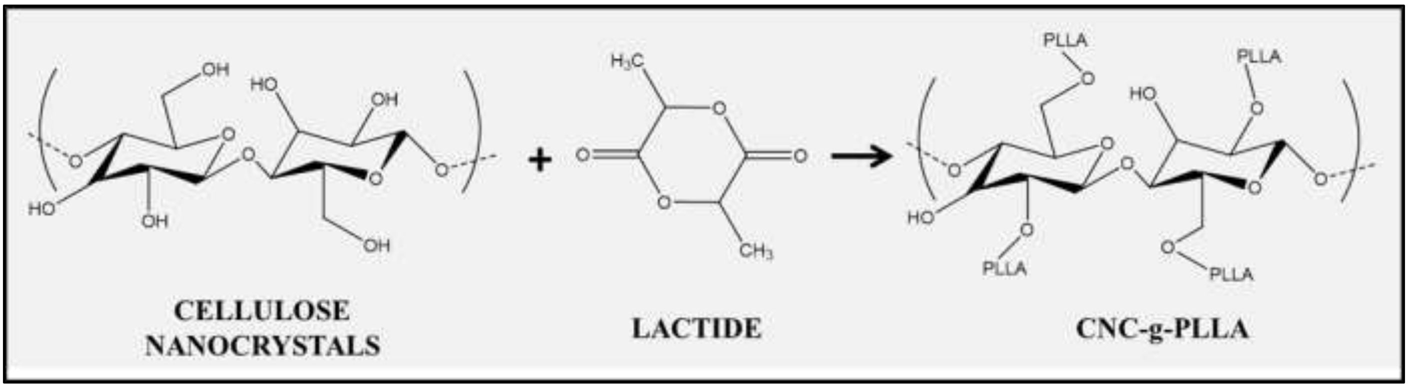

Figure S4. CNC surface chemical modification by grafting PLLA chains onto the CNC surface by ring-opening polymerization (ROP).
